# Immune cell related signature predicts prognosis in esophageal squamous cell carcinoma based on single-cell and bulk-RNA sequencing

**DOI:** 10.3389/fonc.2024.1370801

**Published:** 2024-06-06

**Authors:** Xian Wang, Wei Peng, Yali Zhao, Jiming Sha, Na Li, Shan Huang, Hua Wang

**Affiliations:** ^1^ Department of Pathology, The Second Affiliated Hospital of Anhui Medical University, Hefei, China; ^2^ Department of Pathology, Anhui Medical University, Hefei, China; ^3^ Department of Cardiothoracic Surgery, The Second Affiliated Hospital of Anhui Medical University, Hefei, China; ^4^ Department of Oncology, The Second Affiliated Hospital of Anhui Medical University, Hefei, China; ^5^ Department of Gastroenterology, The Second People’s Hospital of Hefei, Hefei, China

**Keywords:** esophageal squamous cell carcinoma, immune cell infiltration, enrichment analysis, risk model, immune checkpoint, somatic mutation

## Abstract

**Background:**

It has been reported that tumor immune microenvironment performs a vital role in tumor progress. However, acting mechanism of immune cell related genes (IRGs) in esophageal squamous cell carcinoma (ESCC) is uncertain.

**Methods:**

TCGA-ESCC, GSE23400, GSE26886, GSE75241, and GSE196756 datasets were gained via public databases. First, differentially expressed genes (DEGs) between ESCC and control samples from GSE23400, GSE26886, and GSE75241 were screened out by differential expression analysis, and overlapping DEGs were identified. Single-cell transcriptome data of GSE196756 were applied to explore immune cells that might be involved in regulation of ESCC. Then, weighted gene co-expression network analysis was applied to screen IRGs. Next, differentially expressed IRGs (DE-IRGs) were identified by overlapping IRGs and DEGs, and were incorporated into univariate Cox, least absolute shrinkage and selection operator, and multivariate Cox to acquire prognosis-related genes, and ESCC samples were grouped into high-/low-risk groups on the basis of median risk score. Finally, the role of prognosis model in immunotherapy was analyzed.

**Results:**

Totally 248 DEGs were yielded by overlapping 3,915 DEGs in GSE26886, 459 DEGs in GSE23400, and 1,641 DEGs in GSE75241. Single-cell analysis found that B cells, dendritic cells, monocytes, neutrophils, natural killer cells, and T cells were involved in ESCC development. Besides, MEred, MEblack, MEpink, MEblue and MEbrown modules were considered as key modules because of their highest correlations with immune cell subtypes. A total of 154 DE-IRGs were yielded by taking intersection of DEGs and genes in key modules. Moreover, CTSC, ALOX12, and RMND5B were identified as prognosis-related genes in ESCC. Obviously, Exclusion and TIDE scores were notably lower in high-risk group than in the other one, indicating that high-risk group was more responsive to immunotherapy.

**Conclusions:**

Through bioinformatic analysis, we identified a prognosis model consisting of IRGs (CTSC, ALOX12, and RMND5B) in ESCC, providing new ideas for studies related to treatment and prognosis of ESCC.

## 1 Introduction

Esophageal cancer has a consistently worse prognosis with a 5-year survival rate of less than 20% because of its high invasiveness (1). It is ranked seventh in global cancer incidence and sixth in mortality (2). According to clinical histology, esophageal cancer can be categorized into esophageal squamous cell carcinoma (ESCC) and esophageal adenocarcinoma (EAC). As the major subtype of esophageal cancer, ESCC originates from squamous epithelial cells and primarily occurs in the upper and middle esophagus (3). Considering the lack of effective biomarkers, many patients are diagnosed with advanced ESCC. Surgical intervention is the primary treatment approach for ESCC; however, it cannot achieve complete relief for individuals with locally advanced tumors (4). In recent years, extensive research has been conducted on ESCC treatment regimens, including radiotherapy, chemotherapy and targeted therapy. Despite these efforts, the 5-year survival rate for ESCC patients is still disappointingly low (5, 6). Additionally, patients with late-stage ESCC often endure significant suffering, such as difficulty in eating and breathing, which is typically difficult to treat. All underpin an urgent need to identify more effective biomarkers for ESCC to optimize clinical diagnosis and treatment.

The tumor immune microenvironment (TIME) is known to play a crucial role in the occurrence, development and treatment outcomes of tumors (7, 8). Against this backdrop, immunotherapy has recently become an effective and safe method of treating tumors (9, 10). Immunotherapy reduces tumor metastasis and recurrence by stimulating specific immune responses to suppress and kill tumor cells (11). Research has demonstrated favorable therapeutic effects of monoclonal antibodies targeting programmed cell death protein 1 (PD-1) or programmed death-ligand 1 (PD-L1) in treating ESCC (12, 13). More importantly, the recently developed single-cell RNA sequencing (scRNA-seq) has been proven to be capable of dissecting heterogeneous tumors and deciphering the interactions between cancer cells and their microenvironment components (14–17).

Therefore, an immune-related prognostic model was constructed using bioinformatic methods, such as single-cell analysis, differential expression analysis, weighted gene co-expression network analysis (WGCNA) and so forth, based on datasets TCGA-ESCC, GSE23400, GSE26886, GSE75241, and GSE196756 from public databases. Additionally, the mechanism of the prognosis-related genes in ESCC was investigated with the help of gene set enrichment analysis (GSEA), immunology and somatic mutations. In this way, the present study could afford profound implications for uncovering the prognosis and treatment of ESCC.

## 2 Materials and methods

### 2.1 Sources of data

The TCGA-ESCC dataset with gene count, fpkm and annotation files, clinical and survival information, and somatic mutation data of samples were acquired via University of California Santa Cruz (UCSC) Xena (http://xena.ucsc.edu/), from 82 cases of ESCC tumor tissues samples (81 ESCC samples had survival information) and 11 cases of paraneoplastic tissues samples. The microarray datasets GSE53622, GSE23400, GSE26886, GSE75241 were collected from Gene Expression Omnibus (GEO) database (https://www.ncbi.nlm.nih.gov/gds) by R package GEOquery (version 2.62.2) (18), containing 60 ESCC samples with gene expression and clinical information in GSE53622, 53 ESCC tumor tissues samples and 53 paraneoplastic tissues samples in GSE23400, 9 ESCC tumor tissues samples and 18 paraneoplastic tissues samples in GSE26886, 15 ESCC tumor tissues samples and 15 ESCC tumor tissues samples in GSE75241. The single-cell transcriptome dataset GSE196756 was also gained via GEO database, which included 3 treatment-naive ESCC samples and 3 control samples for paired adjacent tissues.

### 2.2 Differential expression analysis

Differentially expressed genes (DEGs) between ESCC and control samples from GSE23400, GSE26886, and GSE75241 datasets were respectively mined via R package limma (version 3.50.1) by setting |log_2_FC| > 1 and adj. *P*< 0.05 (19). After that, up-regulated DEGs in three datasets were taken intersection to yield up-regulated DEGs, and down-regulated DEGs in three datasets were overlapped to yield down-regulated DEGs.

### 2.3 Single-cell transcriptome data analysis

The single-cell transcriptome data of GSE196756 was applied to identify immune cells that might be involved in the regulation of the ESCC. First, data preprocessing and normalization were executed via R package Seurat (version 4.1.0) (20) by setting 100< nFeature< 5000, nCount< 20000, and percent.mt< 5%. Moreover, FindVariableFeatures function was utilized to identify the top 2000 highly variable genes in GSE196756, and the results were visualized via LabelPoints function. To determine the number of principal components for optimal clustering, the principal component analysis (PCA) was implemented. Subsequently, the clusters were reclustered utilizing the JackStraw test algorithm, and the ScoreJackStraw function was applied to calculate gene scores at null distribution, and the number of clusters at the inflection point when the decline in standard deviation flattened out was selected. The nearest neighbor graph was computed in accordance with the euclidean distance in PCA space, and UMAP algorithm was utilized for dimensionality reduction. Moreover, the marker genes in each cell cluster with other clusters were found via the FindAllMarkers function, and they were compared with the data in Human Primary Cell Atlas Data using R package SingleR (version 1.831) (21) to get the cell subtypes, and immune cells types.

### 2.4 WGCNA and enrichment analyses

To screen out module genes associated with immune cell types, WGCNA was implemented in TCGA-ESCC dataset samples by R package WGCNA (version 1.70–3) (22). First, infiltration abundance values of 22 immune cells were estimated applying cell type identification by estimating relative subsets of RNA transcripts (CIBERSORT). The subtypes of the immune cells identified in the single-cell analysis were selected out, and the cells with sample number in infiltration less than 70% were excluded. The Wilcoxon-test was employed to examine for discrepancy in the proportions of above cells between ESCC and control samples, and the scores of them were considered as traits for WGCNA to identify key modules. Then, outliers were removed by clustering the samples to guarantee the accuracy of the analysis. A soft threshold was determined for the TCGA-ESCC dataset for determining that the interactions among genes maximally conformed to the scale-free distribution. Subsequently, the dissimilarity coefficient was introduced by calculating the adjacency and similarity among genes, and the systematic clustering tree among genes was acquired accordingly, and modules were screened out in accordance with the criteria of dynamic tree cutting. What’s more, correlation analysis of modules with traits were accomplished, and the module with adj. *P*<0.05 and |Cor| > 0.3 were sifted out as the key modules. Finally, the DEGs and genes in the key modules were overlapped to generate differentially expressed immune cell related genes (DE-IRGs), and they were enrolled in Gene Ontology (GO) and Kyoto Encyclopedia of Genes and Genomes (KEGG) enrichment analyses via R package clusterProfiler (version 4.2.2) (23) with the threshold of adj. *P<* 0.05.

### 2.5 Creation of a prognosis model

According to the survival information of 81 ESCC samples in TCGA-ESCC dataset, univariate Cox was implemented on DE-IRGs to sift out genes that related to the survival of ESCC. After that, the least absolute shrinkage and selection operator (LASSO) analysis of genes acquired in the previous step was applied to yield genes corresponding to lambda min for proportional hazards (PH) hypothesis test. Subsequently, genes satisfying the PH hypothesis test were incorporated into multivariate Cox analysis to acquire prognosis-related genes. Meanwhile, the expression of prognosis-related genes between ESCC and control samples were also analyzed utilizing Wilcox test in the TCGA-ESCC dataset. Then, the risk score for ESCC samples in the TCGA-ESCC dataset was calculated.


$$\text{risk score}=\sum_{\text{i}=1}^{\text{n}}\left(\text{Coefi}*\text{Expi}\right)$$

(where Coefi denoted the correlation coefficient, and Expi denoted the sample expression corresponding to the gene), and ESCC samples were classified into high-/low-risk groups in accordance with the median risk score. Nevertheless, the survival difference between these two groups were compared applying Kaplan-Meier (K-M) survival analysis. The receiver operating characteristic (ROC) curves were plotted with the aim of evaluating prediction accuracy of the prognosis model, and the expressions of prognosis-related genes between high-/low-risk groups were analyzed. Furthermore, the results were validated by the same methods in the GSE53622.

### 2.6 Screening for independent prognostic factors and gene set enrichment analysis

For further investigating the relationship between ESCC patients’ survival and clinical characteristics, a clinical prognostic model was constructed. Based on clinical characteristics (age, gender, pathologic M, pathologic N, and pathologic T), survival information of ESCC samples, and risk score, the survival differences between high-/low-risk groups were compared for each clinical characteristic. In TCGA-ESCC dataset, risk score and clinical characteristics were analyzed by univariate Cox analysis, and factors meeting P< 0.05 were extracted for PH hypothesis test. The factors satisfying the PH hypothesis test were enrolled in multivariate Cox analysis to sift out independent prognostic factors. After that, a nomogram was created via R package rms (version 6.2–0) (24) to predict the probability of patient survival, and calibration curve was drawn to further validate the results. Ultimately, the GSEA for the prognosis model was also applied by R package clusterProfiler (version 4.2.2) (23) by setting adj. *P*< 0.05 and |NES| > 1 to explore the biological functions and pathways (Background gene set: c2.cp.kegg.v7.4.symbols.gmt).

### 2.7 Immune microenvironment analysis

To assess the association of Major Histocompatibility Complex (MHC) genes with prognosis-related genes, the correlation analysis was carried out. According to the risk score of ESCC samples in TCGA-ESCC dataset and the expressions of 22 MHC genes, correlation coefficients were computed between MHC genes and risk score, and between MHC genes and prognosis-related genes by R package stats (version 4.1.0) (25) by setting |Cor| > 0.3 and *P*< 0.05, respectively. Then, to understand the response to immunotherapy in patients with different risks, the differences in immunotherapy response between high-/low-risk groups was analyzed. Tumor immune dysfunction and exclusion (TIDE), Dysfunction, and Exclusion scores of ESCC samples were computed via TIDE website (http://tide.dfci.harvard.edu), and their differences between high-/low-risk groups and their correlation with risk score were analyzed. After that, patients with TIDE score ≥ 0 were considered deemed to be non-responders to immunotherapy, and those with a TIDE score ≤ 0 were deemed to be responders to immunotherapy, followed by calculation of their proportions in the different risk groups. The correlations of these three scores with risk score were also analyzed utilizing R package stats (version 4.1.0) (25). Furthermore, the association between 48 immune checkpoints and risk score was explored, and expressions of immune checkpoints between different risk groups were also compared.

### 2.8 Somatic mutation analysis

In order to clarify the difference and association of somatic mutations between two risk groups, the somatic mutation data for ESCC samples in TCGA-ESCC dataset was yielded via R package TCGAmutations (version 0.3.0) (26). First, the top 25 genes in the high-/low-risk groups were analyzed for mutual exclusion and co-occurrence characteristics, as well as the mutation frequencies of genes in 10 oncogenic pathways. The top 10 genes with the most mutations in the samples from the high-/low-risk groups were visualized utilizing R package maftools (version 2.10.5) (27). Afterwards, mutual exclusion and co-mutagenesis analysis was applied to obtain important information about disease-associated genes and aberrant pathways. Finally, the OncogenicPathways function was utilized to count the frequency of mutations in the 10 oncogenic pathways in different groups to determine the key oncogenic pathways.

### 2.9 Reverse transcription quantitative real-time polymerase chain reaction

Five ESCC tumor tissues and para-carcinoma tissues from 5 ESCC patients were obtained from The Second Affiliated Hospital of Anhui Medical University with their knowledge and consent. This research was allowed by Ethics Committee of the Second Affiliated Hospital of Anhui Medical University (Approval No.: YX2023–210). In short, total RNA of 10 samples were abstracted through TRIzol in accordance with the manufacturer’s direction. In the back of quality control by Nano drop and NanoPhotometer N50, reverse transcription was implemented using SureScript-First-strand-cDNA-synthesis-kit (Servicebio, Wuhan, China) on the basis of the manufacturer’s amplification. Next, 2xUniversal Blue SYBR Green qPCR Master Mix was utilized to perform qPCR analysis. **Table 1** listed the primer sequences for qPCR. The expression level of prognosis-related gene was counted via 2^−ΔΔCt^ method with GAPDH as the internal reference gene.

**Table 1:** The primers of prognosis-related gene and GAPDH for RT-qPCR.

Gene	Primer
ALOX12 F	TCTGGAGATGGCCCTCAAAC
ALOX12 R	GAAGCTCTTCCATCCCCGAG
CTSC F	AGAGCATCTGTTGAGGGACTCT
CTSC R	CTGCCTTGGAGGTAGGTCAC
RMND5B F	GGGAGTTGCTCGGACTCAAA
RMND5B R	AGAGAGGGTGGCTGAGAGAG
GAPDH F	CGAAGGTGGAGTCAACGGATTT
GAPDH R	ATGGGTGGAATCATATTGGAAC

### 2.10 Immunohistochemistry

Immunohistochemistry was utilized to detect protein expression of prognosis-related genes in ESCC tumor tissues. Initially, tumor tissues were fixed in a 4% paraformaldehyde solution and embedded in paraffin, followed by sectioning. Subsequently, paraffin sections underwent dewaxing, rehydration, and antigen retrieval. Endogenous peroxidase activity was then inactivated by incubating at room temperature for 10 minutes with peroxidase blocker. Sections were incubated with primary antibody overnight, followed by exposure to secondary antibody for 20 minutes at room temperature the next day. Finally, staining was performed using diaminobenzidine (DAB), with counterstaining achieved using hematoxylin.

## 3 Results

### 3.1 Selection of DEGs

**Figure 1:**
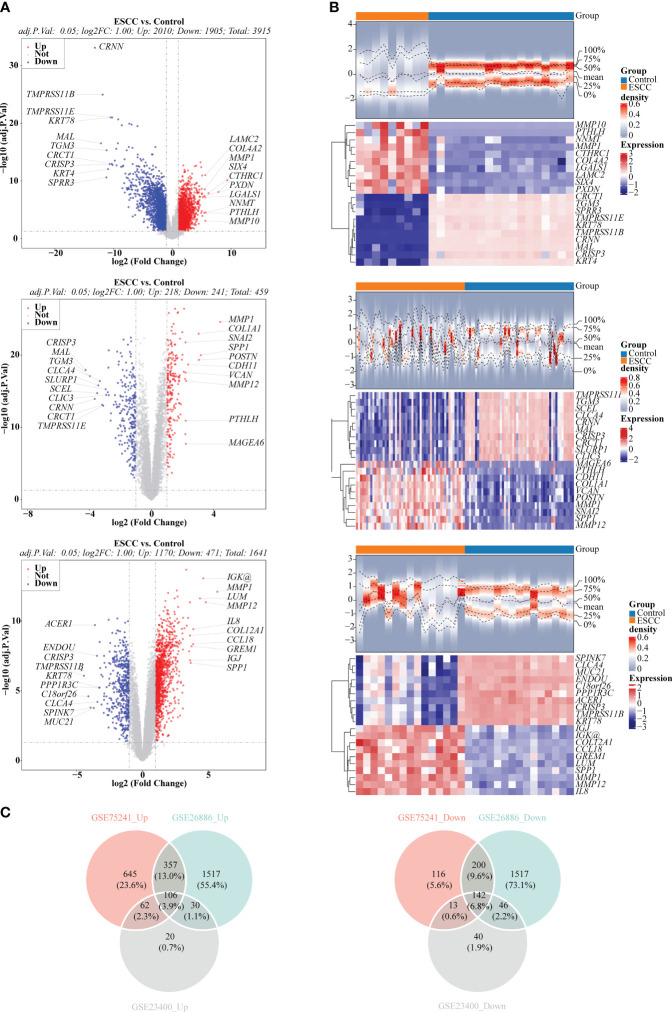
Selection of differentially expressed genes (DEGs) between ESCC and control samples. **(A)** Volcano plots depicted DEGs in GSE26886, GSE23400 and GSE75241 dataset, respectively. Red: upregulation; blue, downregulation. **(B)** Heatmaps depicted DEGs in GSE26886, GSE23400 and GSE75241 dataset, respectively. **(C)** Venn diagrams revealed 106 up-regulated overlapping DEGs and 142 down-regulated overlapping DEGs, respectively.

There were 3,915 DEGs in GSE26886 (**Figures 1A, B**), 459 DEGs in GSE23400 (**Figures 1A, B**), and 1,641 DEGs in GSE75241 (**Figures 1A, B**), respectively, and the heat maps illustrated the top10 up- and down- regulated DEGs in three datasets. Subsequently, 106 up-regulated and 142 down-regulated DEGs were yielded by taking intersection of up-regulated DEGs and down-regulated DEGs in three datasets, respectively, and they were merged to get 248 DEGs (**Figure 1C**; **Supplementary Table S1**).

### 3.2 B cells, dendritic cells, monocytes, neutrophils, natural killer cells and T cells were annotated in GSE196756

**Figure 2:**
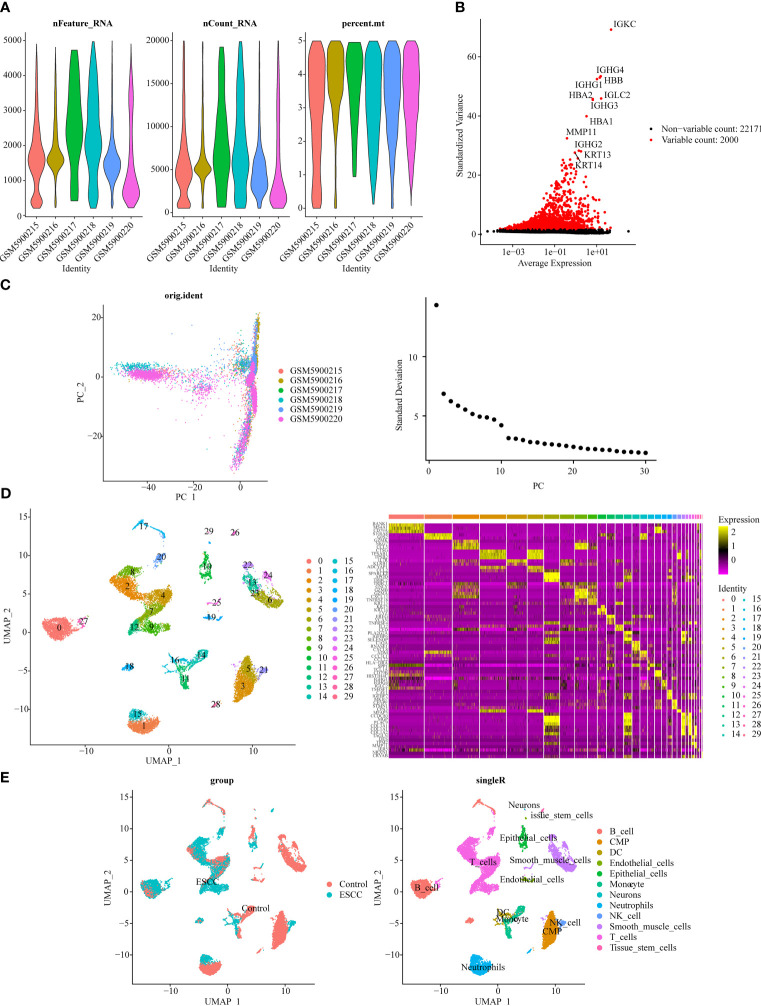
Single-cell transcriptome data analysis for GSE196756. **(A)** Violin plot of nFeature, nCount, and percent.mt after quality control. **(B)** Volcano plot depicted 2,000 highly variable genes (red). **(C)** Principal component analysis (PCA) determined the optimal dimensions. **(D)** UMAP clustering plot displayed 30 cell clusters in the sample, and heat map depicted the marker genes in 30 cell clusters. **(E)** UMAP clustering plot revealed 12 different cell subtypes.

The violin plot demonstrated the nFeature, nCount, and percent.mt in different samples after quality control (**Figure 2A**). Besides, volcano plot illustrated the top 20 highly variable genes, including IGKC, IGHG1, HBA1, etc. (**Figure 2B**). From **Figure 2C**, it could be seen that when the number of clusters was 20, the standard deviation decreased gently, thus it was taken as the optimal number of clusters. When resolution = 0.8, the 30 cell clusters were yielded (**Figure 2D**). Eventually, 12 cell subtypes were finally identified, and which included 6 immune cells (B cells, dendritic cells, monocytes, neutrophils, natural killer cells, T cells) (**Figure 2E**).

### 3.3 Screening for the key modules

**Figure 3:**
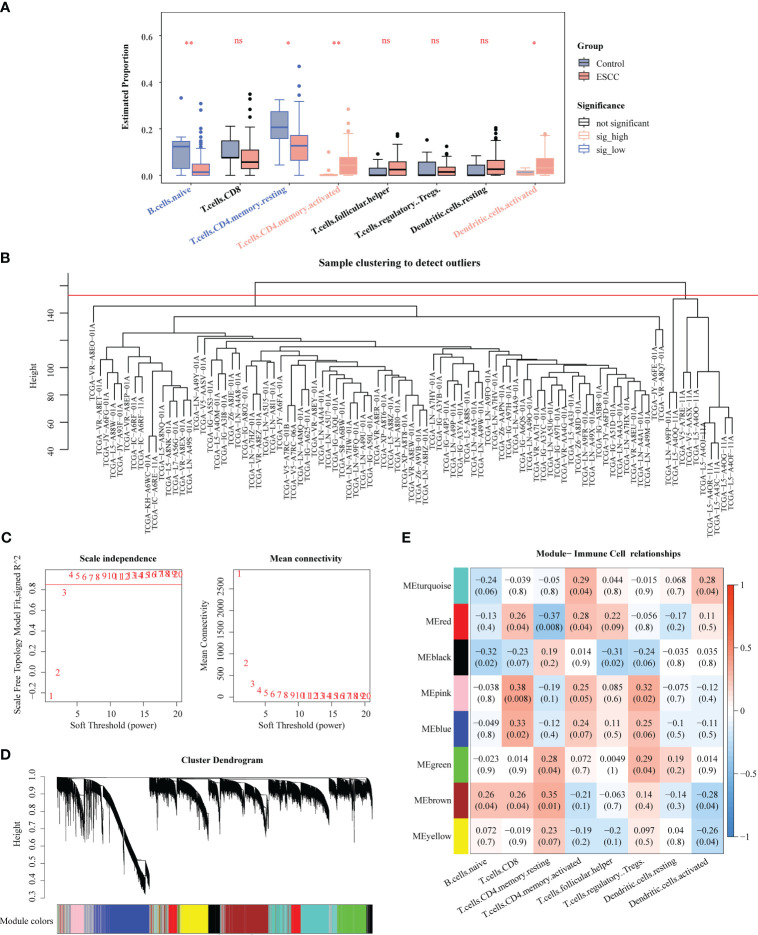
Identification of key genes associated with immune cells by WGCNA. **(A)** Comparison of immune cells between ESCC and control samples. **(B)** Clustering of TCGA-ESCC dataset samples. **(C)** Determination of the optimal soft threshold. **(D)** All genes were classified into various modules by hierarchical clustering. **(E)** Heatmap of module-trait correlation. * *P* < 0.05, ** *P* < 0.01; ns, no significance.

Totally 8 immune cell subtypes were identified form TCGA-ESCC (naive B cells, CD8 T cells, memory resting CD4 T cells, memory activated CD4 T cells, follicular helper T cells, regulatory T cells (Tregs), resting dendritic cells, activated dendritic cells), among which the proportions of naive B cells, memory resting CD4 T cells, memory activated CD4 T cells, and activated Dendritic cells were notably different between ESCC and control samples, suggesting that the development and progression of ESCC was related to these immune cells (**Figure 3A**). **Figure 3B** manifested a better overall clustering of the dataset samples without eliminating samples. Based on the location of red line in **Figure 3C**, the soft threshold of 4 was determined. At this point, the network was approaching the scale-free distribution and presented a flat trend, as seen by the vertical coordinate R^2^ exceeding 0.85 and the mean value of the adjacency function steadily approaching 0. Next, the 8 modules were finally sifted out by building the co-expression matrix (**Figure 3D**), among which the MEred, MEblack, MEpink, MEblue, and MEbrown modules were considered as the key modules because their correlations with immune cell subtypes score conformed to adj. *P<*0.05 and |Cor| > 0.3, and which totally contained 8,630 genes (**Figure 3E**).

### 3.4 DE-IRGs were related to skin formation related pathways

**Figure 4:**
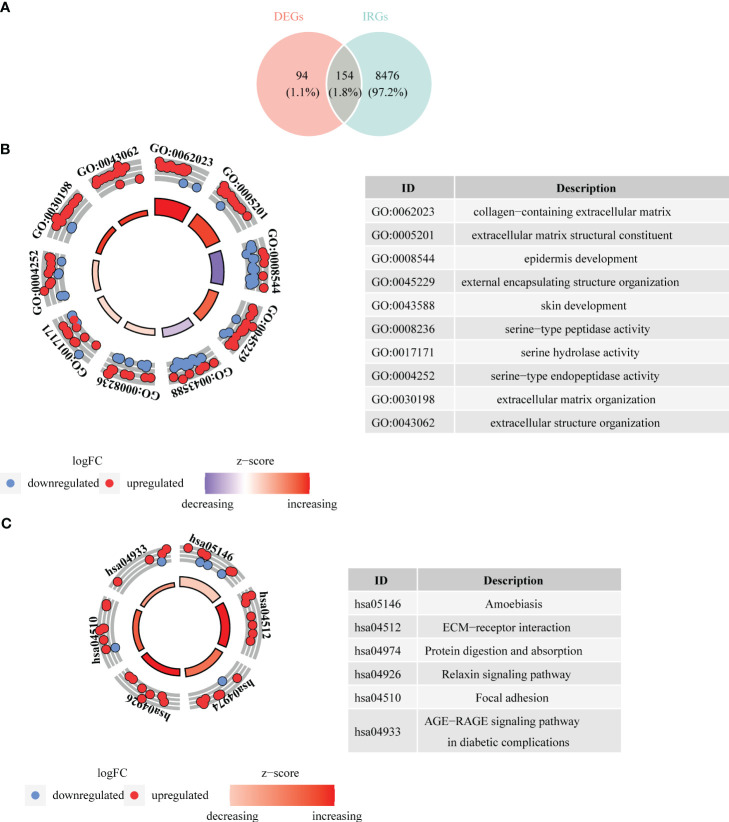
Acquisition of differentially expressed immune cell related genes (DE-IRGs) and enrichment analysis. **(A)** Venn diagrams revealed 154 DE-IRGs. **(B)** Bagua plot of GO analysis on DE-IRGs. **(C)** Bagua plot of KEGG analysis on DE-IRGs.

Taking intersection of DEGs, and genes in key modules resulted in 154 DE-IRGs (**Figure 4A**). Besides, DE-IRGs were involved in GO entries containing epidermis development, skin development, extracellular matrix organization, collagen metabolic process, epidermal cell differentiation, etc. (**Figure 4B**). Simultaneously, they were also enriched to KEGG pathways like ECM-receptor interaction, protein digestion and absorption, relaxin signaling pathway, AGE-RAGE signaling pathway in diabetic complications (**Figure 4C**).

### 3.5 The prognostic model demonstrated an excellent ability for predicting ESCC patients

**Figure 5:**
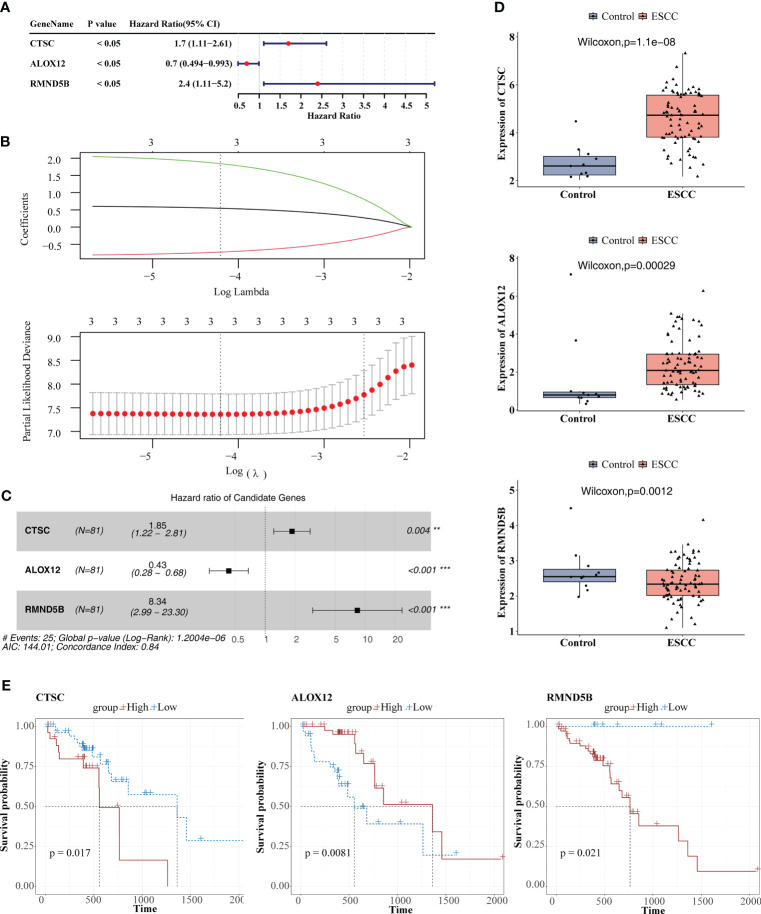
Identification of prognosis-related genes. **(A)** Univariate cox analysis. **(B)** Least absolute shrinkage and selection operator (LASSO) regression analysis. **(C)** Multivariate Cox analysis. **(D)** Comparison of the expression of the three prognosis-related genes between ESCC and control samples. **(E)** KM survival curves between high and low expression groups of three prognosis-related genes. ** *P* < 0.01, *** *P* < 0.001.

**Figure 6:**
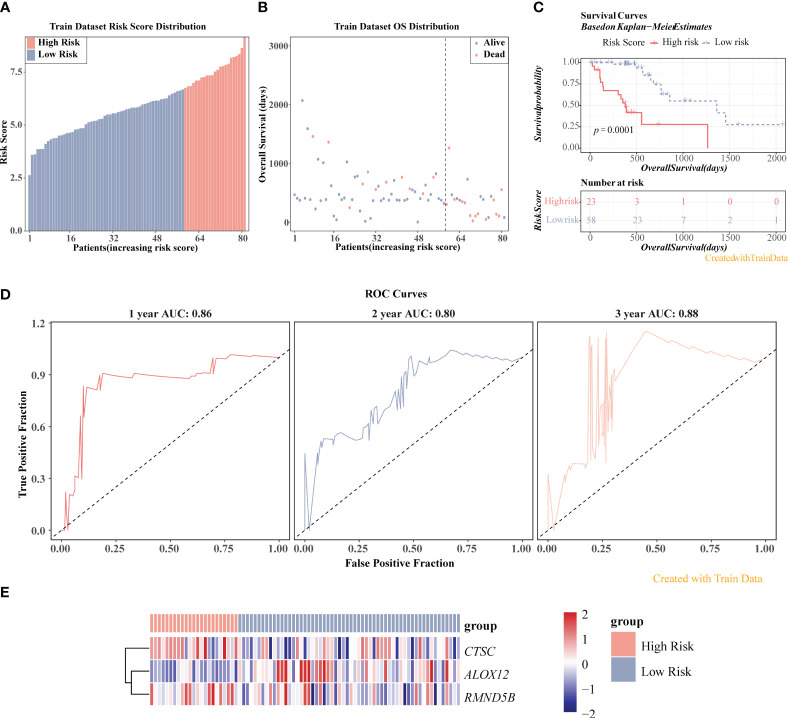
Prognostic value of three prognosis-related genes in TCGA-ESCC dataset. **(A)** Risk score distribution of ESCC samples. **(B)** Overall survival (OS) distribution of ESCC samples. **(C)** K-M survival analysis of ESCC samples. **(D)** ROC curves for 1-, 2-, and 3-year survival in ESCC patients. **(E)** Heatmap displayed the expression levels of three genes in high-/low-risk groups.

The three prognosis-related genes, namely CTSC, ALOX12, and RMND5B were finally identified via univariate Cox, LASSO, PH hypothesis test and multivariate Cox (**Figures 5A–C**), and their expressions were all notably different between ESCC and control samples (**Figure 5D**). It could be found that the hazard ratio (HR) of ALOX12 was less than 1, but its expression was higher in ESCC than in normal tissues, while RMND5B showed the opposite trend. It may be due to the fact that the high expression of the gene is to counteract a certain aberration in the tumor cells, or it may be due to the fact that the gene exerts its anti-tumor effect by affecting the immune infiltrating cells. Meanwhile, we found that patients in the CTSC and RMND5B high expression groups had a worse prognosis by the K-M survival curve, while patients in the ALOX12 high expression group had a better prognosis (**Figure 5E**). **Figure 6A** showed that the risk scores of TCGA-ESCC dataset samples were all greater than 0. From **Figure 6B**, it could be seen that the survival samples were mainly clustered in the regions with lower risk score, while the death samples were opposite. Besides, the K-M curve showed that the survival of high-risk group was substantially lower than the other one (**Figure 6C**). Undoubtedly, the area under curve (AUC) values of 1- (AUC = 0.86), 2- (AUC = 0.80) and 3- (AUC = 0.88) year were all greater than 0.8, indicating that the prognosis model had an excellent ability of predicting ESCC patients (**Figure 6D**). From the heatmap, it was clear that ALOX12 was lowly expressed in high-risk group, while the opposite was true for CTSC, and RMND5B (**Figure 6E**). The results were further validated in the GSE53622 (**Supplementary Figure S1**). KM curve suggested that high-risk patients demonstrated a terrible survival rate (*P*=0.0408), as well as AUC values were 0.67, 0.67, and 0.62 for 1-, 2-, and 2-years, correspondingly. These findings indicated that prognostic model had reliable generalization in predicting ESCC survival.

### 3.6 The clinical prognostic model had a favourable ability for predicting ESCC patients, and the prognosis model was related to immune related pathways

**Figure 7:**
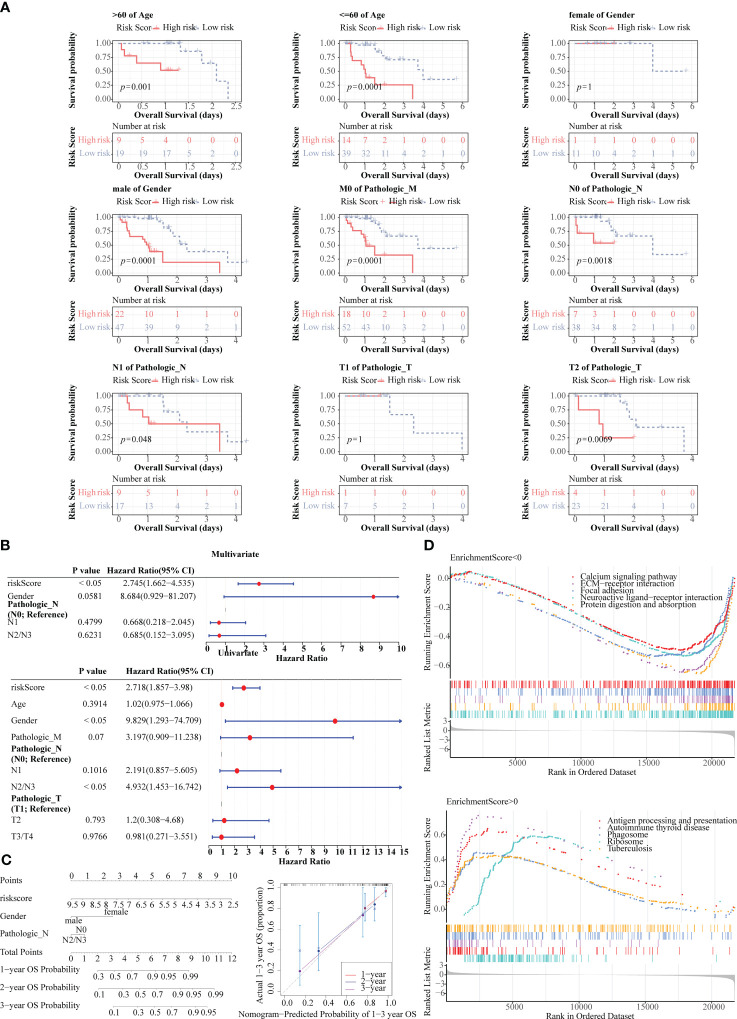
Prognostic value of risk score and clinical characteristics in TCGA-ESCC dataset. **(A)** K-M survival analysis between different clinical subgroups. **(B)** Univariate and multivariate Cox analysis of risk score and clinical characteristics. **(C)** Nomogram and calibration curve for predicting 1-, 2-, and 3-year survival in ESCC patients. **(D)** Gene set enrichment analysis (GSEA) for the prognosis model.

K-M curves demonstrated notable survival differences between two risk groups in age, male, pathologic M0, pathologic N0, pathologic N1, and pathologic T2 (**Figure 7A**). Additionally, three independent prognostic factors, namely risk score, gender, and pathologic N, were finally gained through univariate and multivariate Cox (**Figure 7B**). Subsequently, nomogram revealed that the clinical prognostic model had a favorable ability for predicting ESCC patients, and this result was further confirmed through calibration curve (**Figure 7C**). What’s more, the prognosis model was enriched to 74 KEGG pathways, among which the pathways with positive enrichment score included antigen processing and presentation, phagosome, allograft rejection, natural killer cell mediated cytotoxicity, oxidative phosphorylation (**Figure 7D**), and the pathways that with negative enrichment score contained neuroactive ligand-receptor interaction, protein digestion and absorption, signaling pathways regulating pluripotency of stem cells, calcium signaling pathway, etc. (**Figure 7D**).

### 3.7 The prognosis model played an essential role in the immunotherapy of ESCC

**Figure 8:**
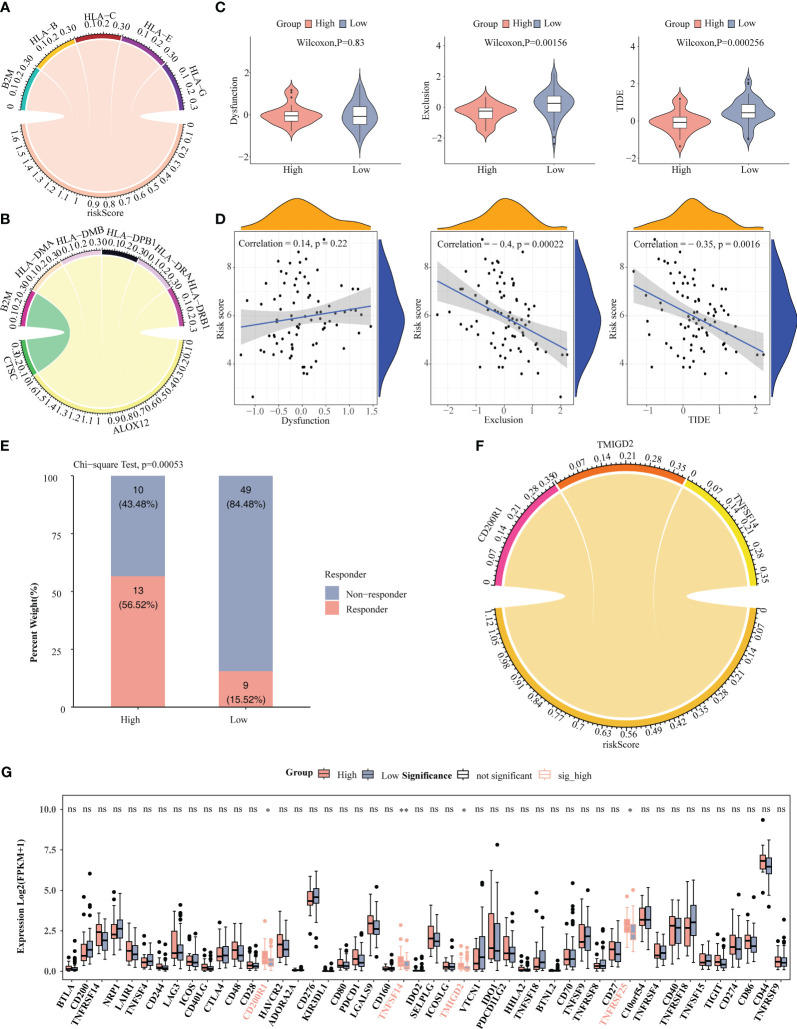
Correlation analysis of risk score with immune microenvironment. **(A)** Chord plot depicted five MHC genes positively associated with risk score. **(B)** Chord plot depicted correlation between five MHC genes and three prognosis-related genes. **(C)** Comparison of TIDE, Dysfunction, and Exclusion scores between high-/low-risk groups. **(D)** Correlation analysis of risk scores with TIDE, Dysfunction, and Exclusion scores. **(E)** Comparison of the number of responders and non-responders to immunotherapy between high-/low-risk groups. **(F)** Correlation analysis of risk score with immune checkpoints. **(G)** Comparison of immune checkpoints between high-/low-risk groups. * *P* < 0.05, ** *P* < 0.01; ns, no significance.

As can be seen from the chord plot, five MHC genes (B2M, HLA-B, HLA-C, HLA-E, and HLA-G) were positively correlated with risk score (**Figure 8A**). Besides, there was a positive correlation between B2M and CTSC, whereas HLA-DMA, HLA-DMB, HLA-DPB1, HLA-DRA, and HLA-DRB1 all had negative correlations with ALOX12 (**Figure 8B**). Violin plots showed that the Exclusion and TIDE scores were notably lower in high-risk group than in the other one, manifesting that samples in high-risk group were more responsive to immunotherapy, and that immune cells penetrated into the tumor tissues more easily (**Figure 8C**). Meanwhile, the Exclusion and TIDE scores were negatively correlated with the risk score, indicating that the higher the risk score, the lower the degree of immune rejection of the patients and the more sensitive response to immunotherapy (**Figure 8D**). As demonstrated in **Figure 8E**, there was a marked difference in the number of immunotherapy responders and non-responders between high-/low-risk groups. Apparently, there were associations between risk score and the immune checkpoints CD200R1, TMIGD2 and TNFSF14 (**Figure 8F**). Box plot revealed that the expressions of CD200R1, TNFSF14, TMIGD2, and TNFRSF25 were notably higher in high-risk group than in the other one, demonstrating that their associated immune pathways or activities were more active in high-risk group (**Figure 8G**).

### 3.8 The prognosis model might influence ESCC patients prognosis by modulating TP53 and NOTCH pathways

**Figure 9:**
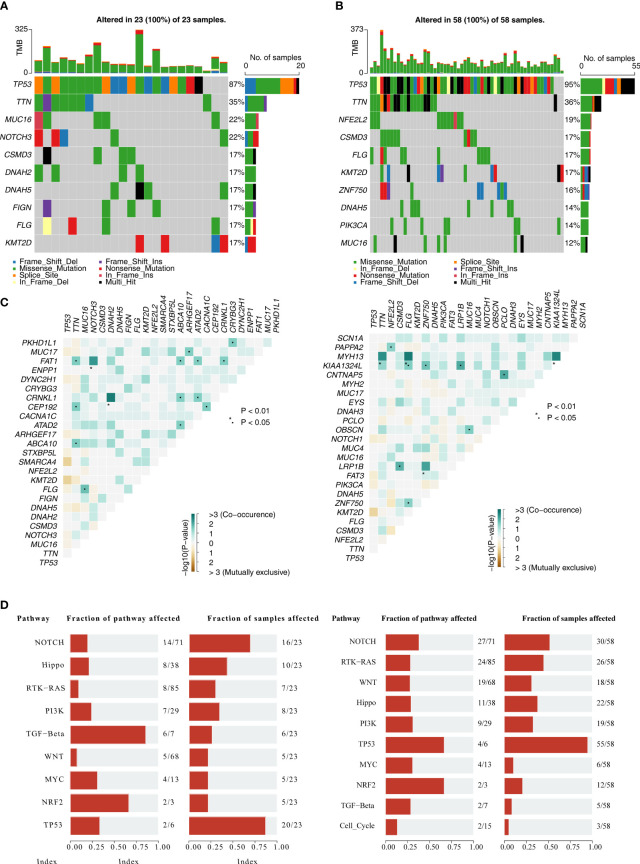
Gene mutation analysis. **(A)** Waterfall plot of mutations in high-risk groups. **(B)** Waterfall plot of mutations in low-risk groups. **(C)** Heatmap revealed top 25 mutated genes in high-/low-risk group. **(D)** Mutation frequency analysis of 10 oncogenic pathways in high-/low-risk group.

As can be seen from the waterfall plots, 10 genes were mutated in high-risk group samples, with the highest number of mutated samples was missense mutation, and TP53 had the highest mutation frequency, followed by TTN (**Figure 9A**), TP53, and TTN also had the highest mutation frequency in low-risk group (**Figure 9B**). Thereafter, heatmap showed a higher number of co-mutations occurring among the top 25 mutated genes in high-/low-risk groups, suggesting that these mutations might occur simultaneously in the same patient and that these genes might be involved in interdependent pathways or functions (**Figure 9C**). Moreover, pathway with the highest frequency of gene mutations in high-risk group was TGF-Beta, and TP53 had the highest number of samples with pathway mutations (20 cases with mutations), whereas the pathway with the highest frequency of gene mutations in low-risk group was TP53 and NRF2, and the pathway with the highest number of samples with pathway mutations was TP53 (55 cases with mutations). Therefore, the variability of gene mutations within the TP53 and NOTCH carcinogenic pathways might be strongly associated with patient risk score (**Figure 9D**).

### 3.9 Expression verification of CTSC, ALOX12 and RMND5B by RT-qPCR and immunohistochemistry

**Figure 10:**
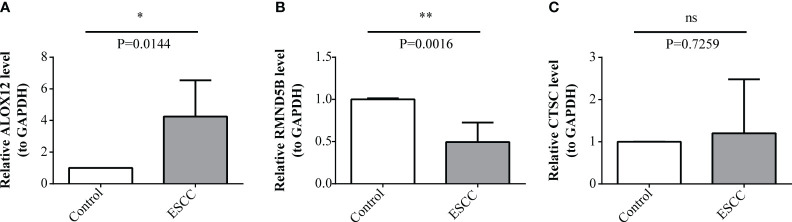
Expression levels validation of prognosis-related genes through RT-qPCR. **(A-C)** Comparison of ALOX12 **(A)**, RMND5B **(B)**, and CTSC **(C)** expression levels between ESCC and control sample. * *P* < 0.05, ** *P* < 0.01; ns, no significance.

**Figure 11:**
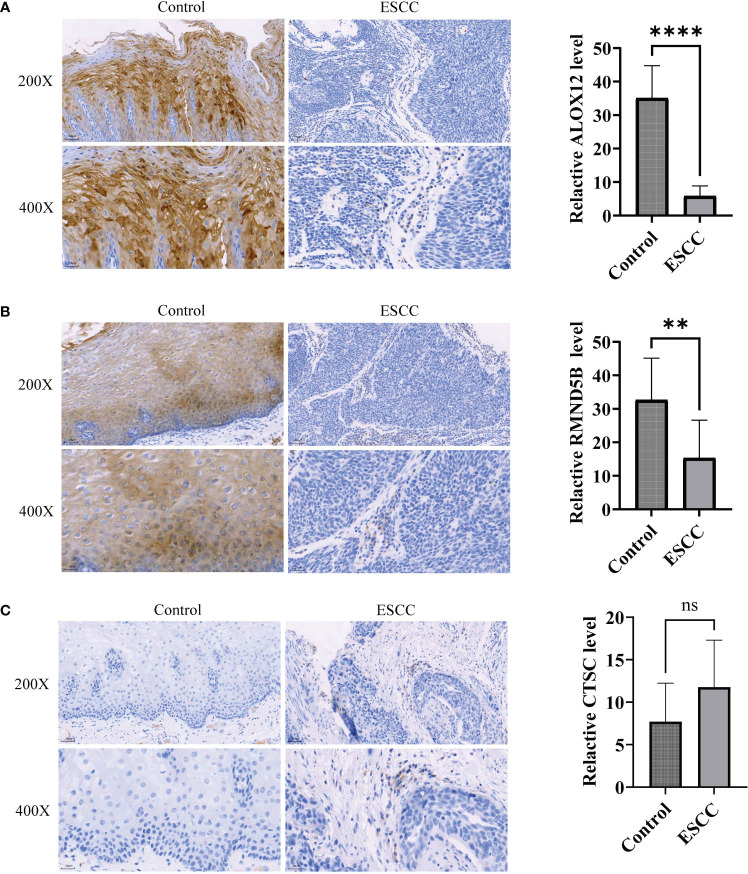
Immunohistochemistry was utilized to detect protein expression of prognosis-related genes in ESCC tumor tissues. **(A-C)** Comparison of ALOX12 **(A)**, RMND5B **(B)**, and CTSC **(C)** expression levels between ESCC and control sample. ** *P* < 0.01, **** *P* < 0.0001; ns, no significance.

At last, the expression of three prognosis-related genes in clinical samples was experimentally validated. The RT-qPCR result revealed that ALOX12 was markedly up-regulated (*P*=0.0144) and RMND5B was markedly down-regulated (*P*=0.0016) in tumor samples compared with controls, while the expression of CTSC was not notably different (*P*=0.7259) between tumor and control samples (**Figures 10A–C**). Afterwards, the protein expression of three prognosis-related genes was further verified by immunohistochemistry. The results demonstrated that the protein expression of ALOX12 and RMND5B was significantly lower in tumor tissues compared to paraneoplastic tissues, while there was no significant difference in the protein expression of CTSC (**Figures 11A–C** ). This might be due to the small sample size or the heterogeneity of the sample, after which we will do further validation.

## 4 Discussion

ESCC is the most common pathological type of esophageal cancer in Asian countries, and it is the fourth leading cause of cancer-related deaths in China (28). Currently, owing to its poor prognosis and limited treatment efficacy, there is a need to develop clinically valuable biomarkers. The immune microenvironment and various immune cells perform pivotal roles in tumor progression and treatment (29). In this research, we constructed a prognostic model on the basis of immune cell–related prognostic biomarkers in the ESCC using public transcriptome data.

Through the identification of immune cell types and subsequent bioinformatic screening, three genes related to ESCC prognosis were identified, including cathepsin C (CTSC), arachidonate 12-lipoxygenase (ALOX12) and required for meiotic nuclear division 5 homolog B (RMND5B). CTSC is a lysosomal cysteine protease of the papain family and is correlated with the development of squamous cell carcinoma (30). Zhao et al. showed that the expression of CTSC gene was increased in myeloid cells of Pancreatic adenosquamous carcinoma (31). Moreover, a recent work by Han et al. revealed that CTSC promotes colorectal cancer metastasis by upregulating CSF1 to regulate immune evasion (32). However, the specific mechanisms of action of CTSC in ESCC occurrence and progression have not been studied. Our analysis of overall survival data showed that CTSC can serve as a prognostic risk factor (hazard ratio [HR] > 1) in patients with ECSS. ALOX12 acts on polyunsaturated fatty acids to produce active lipid molecules and plays a significant role in inflammation and oxidative reactions (33). A previous study by Chen et al. demonstrated that genetic and pharmacological inhibition of ALOX12 can suppress the growth and migration of lung cancer cells, induce apoptosis and increase sensitivity to chemotherapy (34). Furthermore, a research by Weng et al. showed that high ALOX12 expression in colorectal cancer can indicate increased immune infiltration and a better response to immunotherapy (35). Analysis of the overall survival data in ESCC patients showed that ALOX12 is a protective factor for patient survival (HR< 1). Nevertheless, further research is necessary to clarify the specific mechanisms of action of ALOX12 in ESCC progression. RMND5B, also known as GID2, has ubiquitin-related protease activity. Deng et al. found that GID2 interacts with CDKN3 and regulates the growth and apoptosis of pancreatic cancer cells (36). The present study is the first to demonstrate that RMND5B is a prognostic marker for the survival of ESCC patients and is a risk factor (HR > 1). The specific mechanisms of action of RMND5B in ESCC progression have not been reported previously.

Further, we verified the expression of three prognostic genes by RT-qPCR and immunohistochemistry in this study. Previous studies of this project have shown that CTSC and RMND5B are cancer-promoting factors. However, this experiment indicates that RMND5B is actually under-expressed in tumor tissues, suggesting that the activity of this gene may be enhanced in a number of ways (gene, mutations, chromosomal rearrangements, gene amplification, etc.), not necessarily by overexpression. ALOX12 is a tumor suppressor gene, but it is highly expressed in tumor tissues by RT-qPCR, which may be due to the fact that this gene can enhance the occurrence of tumor suppressor pathway through over-expression, thus achieving the function of tumor suppressor. However, immunohistochemical results showed that its expression in tumor tissues was reduced. Because there are many levels of regulation of gene expression, of which regulation at the transcriptional level is only one link, there are also post-transcriptional regulation and post-translational regulation, and post-translational regulation plays a role in the final protein expression. In addition, factors such as mRNA degradation, protein degradation and modified folding may cause differences in mRNA abundance and protein expression levels. Therefore, the specific mechanisms of prognostic genes and tumor development need to be further investigated.

Following the analysis of these three prognosis genes associated with immune of patients with ESCC, an immune-related prognostic model based on these three genes was further constructed. The prognostic model exhibited a marked survival difference between high- and low-risk patients, with high-risk patients having a poorer survival. In addition, the results of the ROC curve confirmed the high reference value of this prognostic model for survival prediction in ESCC patients. Currently, there are widely reports on prognostic models for ESCC patients, such as fibroblast-associated prognostic models, cuproptosis-related prognostic models, cell death-related prognostic models, etc. Compared with these models, the immune-related prognostic model constructed in this study incorporated immune-related genes, which helped to identify patients with a higher risk of disease progression or recurrence, and also captured the complex interactions between ESCC and the immune system accurately. Meanwhile, the constructed immune-related prognostic model could add valuable information about the ESCC’s immune response, thus improving the accuracy of prognostic prediction and contributing to therapeutic decision-making. In addition, by assessing the immune characteristics of patients, the model could guide the selection of appropriate immunotherapies and improve treatment outcomes.

According to the Cox analysis results, the risk score was identified as an independent prognostic factor. A nomogram predicting the 1- to 3-year survival rate of patients with ESCC was created, which incorporated the independent prognostic factors of gender and pathologic N stage. A calibration curve was plotted, and the survival predictions made by this curve model closely matched the actual survival rates of the patient cohort. This further underscores the clinical potential of this curve model.

Moreover, GSEA revealed that the calcium signaling pathway, ECM-receptor interaction and focal adhesion were enriched in low-risk patients. Previous studies have confirmed the association between the calcium signaling pathway and ESCC cell proliferation (37). Various bioinformatics studies have demonstrated that the ECM-receptor interaction is associated with the development and progression of ESCC or EAC (38–41). Focal adhesions have been found to be associated with cell migration, as well as focal adhesion kinases with adhesive signal transduction (42). Moreover, focal adhesion kinases are known to have a pivotal role in cancer progression (42), including ESCC progression (43). In line with these findings, our results also suggest close associations of these pathways and functions with ESCC progression and patient survival.

The treatment strategy of immunotherapy focuses on activating the patient’s immune system to combat cancer, and it has become one of the common approaches for cancer treatment, alongside surgery, radiation therapy and targeted therapy (44). Immune checkpoint molecules are receptor–ligand pairs that regulate immune stimulation or suppression, playing crucial roles in maintaining immune tolerance and reducing tissue damage (45). Immune checkpoint molecules in cancer cells are associated with immune evasion, and targeting these molecules can prevent cancer cells from escaping the immune system, thus enhancing immune recognition. In patients with ESCC, immunotherapy targeting PD-1/PD-L1 immune checkpoint molecules has revealed promising clinical efficacy (46). Our study indicates that based on the risk scoring model, high-risk patients have significantly lower exclusion scores and TIDE scores compared with other patients. This suggests that high-risk patients exhibit a stronger response to immunotherapy, and immune cells can more easily infiltrate tumor tissue. Additionally, the exclusion and TIDE scores showed negative correlations with the risk score, indicating that the degree of immune exclusion decreases as the risk score increases in patients with ESCC, and the response to immunotherapy becomes more sensitive. Our risk-scoring model can provide valuable guidance for clinical decisions regarding immunotherapy for patients. Furthermore, we identified correlations between risk score and the expressions of the immune checkpoint genes CD200R1, TMIGD2 and TNFSF14. The expression levels of CD200R1, TNFSF14, TMIGD2 and TNFRSF25 were markedly higher in high-risk patients than in low-risk patients, suggesting that related immune pathways or activities are more active in high-risk patients. Recently, a CD200R1-targeting antibody, 23ME-00610, was reported to enhance the function of anti-tumor T cells by blocking CD200:CD200R1 binding, thus inhibiting tumor growth while participating in the immune activation pathway (47). TNFSF14, also known as LIGHT, is highly effective in driving anti-tumor immune responses and inducing changes in the tumor microenvironment (48). Studies have indicated that incorporating LIGHT in immunotherapy regimens is highly promising (48), and future research is warranted. Our results suggest that CD200R1 and TNFSF14 are potential targets for ESCC immunotherapy.

In conclusion, our study identified three prognosis related biomarkers associated with immune cells in patients with ESCC, offering fresh insights into the mechanisms of the immune microenvironment and ESCC progression. The risk-scoring model based on these prognostic biomarkers offers future directions for understanding ESCC progression. However, our research has certain limitations. Firstly, the sample size and information from the public database were limited, and the clinical applicability of the nomogram needs validation with data from a greater number of clinical patients. Second, the roles of these three prognostic biomarkers in the development and progression of ESCC require further investigation, and their relationships with the immune microenvironment need to be explored further. We will continue to monitor the progress of research related to these three biomarkers and their associations with the tumor immune microenvironment in ESCC.

## Data availability statement

The original contributions presented in the study are included in the article/**Supplementary Material**. Further inquiries can be directed to the corresponding authors.

## Ethics statement

The studies involving humans were approved by Ethics Committee of the Second Affiliated Hospital of Anhui Medical University (Approval No.: YX2023-210). The studies were conducted in accordance with the local legislation and institutional requirements. The participants provided their written informed consent to participate in this study.

## Author contributions

XW: Conceptualization, Data curation, Methodology, Project administration, Writing – original draft. WP: Conceptualization, Methodology, Writing – review & editing. YZ: Data curation, Writing – review & editing. JS: Formal Analysis, Writing – review & editing. NL: Writing – review & editing. SH: Writing – review & editing. HW: Conceptualization, Data curation, Methodology, Project administration, Writing – original draft, Writing – review & editing.
