# Supplementary material for: Immune cell related signature predicts prognosis in esophageal squamous cell carcinoma based on single-cell and bulk-RNA sequencing
Source: Front Oncol. 2024 Jun 6;14:1370801. doi: 10.3389/fonc.2024.1370801 (PMC11187079; doi:10.3389/fonc.2024.1370801)
Supplement: Supplementary file 1 [file DataSheet_1.docx]

Supplementary Material

# Supplementary Figures and Tables

## Supplementary Figures


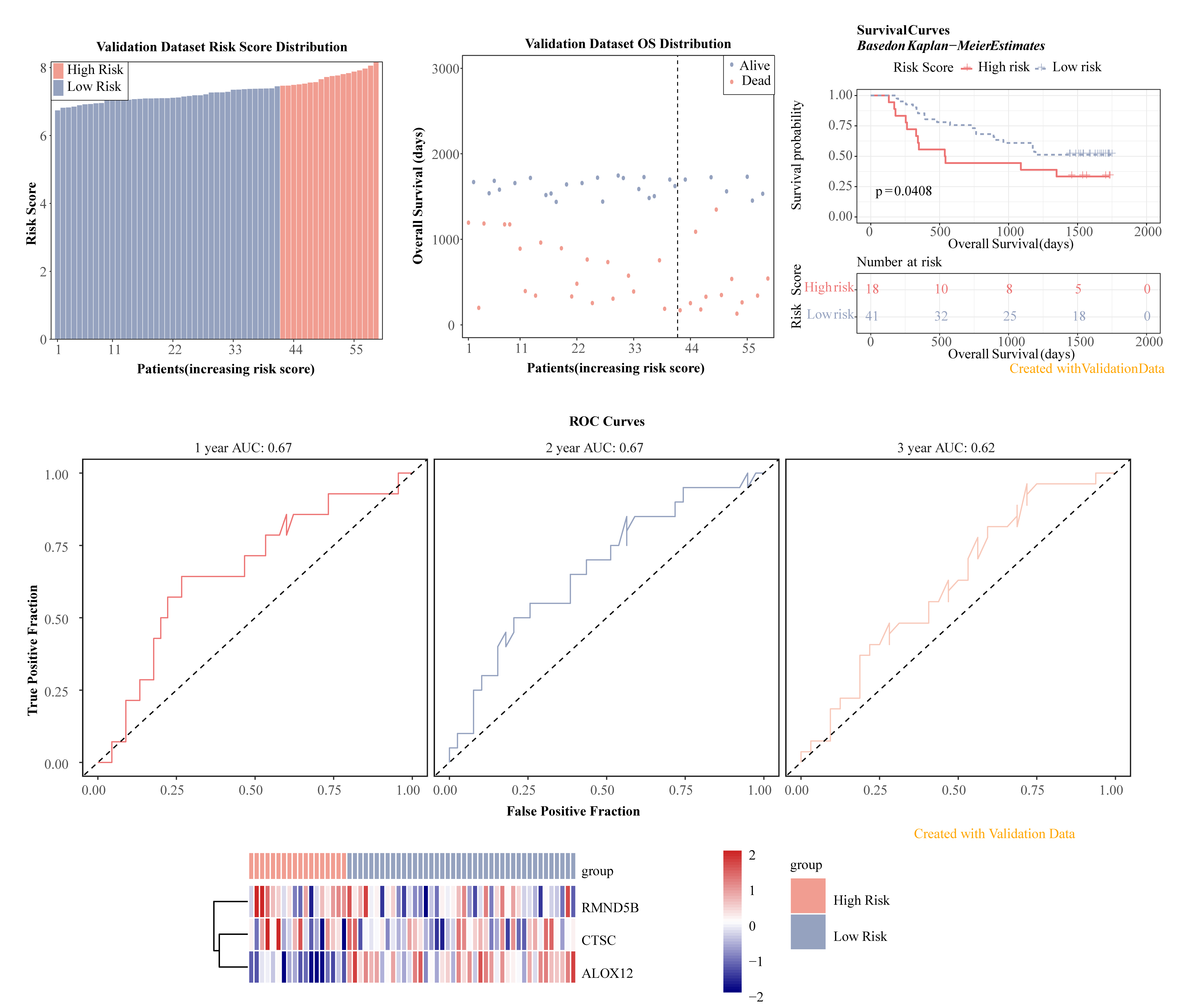


**Supplementary Figure 1.** Prognostic value of three prognosis-related genes in GSE53622 dataset
